# Results From an All Wales Trainee Led Collaborative Prospective Audit on Management of Ankle Fractures

**DOI:** 10.7759/cureus.19269

**Published:** 2021-11-05

**Authors:** Sandeep Gokhale, Prashanth D'sa, Rishi Agarwal, Juliet Clutton, Kunal Roy, Eleanor Clare Carpenter, Khitish Mohanty, Paul Hodgson

**Affiliations:** 1 Trauma and Orthopedics, University Hospital of Wales, Cardiff, GBR; 2 Trauma and Orthopedics, Wrexham Maelor Hospital, Wrexham, GBR

**Keywords:** ankle trauma, weight bearing, compliance, ankle fracture management, boast guidelines

## Abstract

Aim

The purpose of this all Wales national audit was to compare compliance against British Orthopedic Association Standards for Trauma (BOAST) guidelines on the management of ankle fractures.

Methods

A multi-center prospective audit of the management of adult ankle fractures was conducted between February 2, 2020, and February 17, 2020, via the Welsh Orthopedic Research Collaborative (WORC). Regional leads were recruited in nine NHS hospitals across six university health boards, and recruited collaborators in their respective hospitals. Questionnaires for the data collection on both surgical and conservative management were made available via a password-protected website (walesortho.co.uk). We defined early weight-bearing (EWB) as unrestricted weight-bearing on the affected leg within three weeks of injury or surgery and delayed weight-bearing (DWB) as unrestricted weight-bearing after three weeks of injury or surgery.

Results

A total of 28 collaborators contributed data for 238 ankle fractures. Poor documentation at the time of injury was noted. Less than 50% of patients with posterior malleolus fracture had a CT scan for further evaluation. Eighty-four percent of the non-operatively treated patients did not have a weight-bearing X-ray (WBXR). Patients who had a WBXR were more likely to be allowed EWB but this was not statistically significant. EWB was allowed in 59.43% and 10% of the non-operatively and operatively treated patients, respectively. DWB was higher in patients who had fixation of the posterior malleolus or syndesmosis.

Conclusion

There is poor compliance with BOAST guidelines on the management of ankle fractures across Wales. We need to improve documentation and also consider performing a CT scan when the posterior malleolus is fractured. A weight-bearing X-ray should be performed more often to ascertain the stability of an ankle fracture, and those that are deemed stable should be treated with early weight-bearing. The guidelines need to be clearer regarding weight-bearing after fixation especially when posterior malleolus and/or syndesmosis are fixed.

## Introduction

Ankle fracture is one of the commonest lower limb injuries and, with increasing incidence, they are expected to cost the NHS about 2.2 billion pounds per annum in 2020 [1,2]. In the United Kingdom, the British Orthopedic Association (BOA) and the British Orthopedic Foot and Ankle Society (BOFAS) have suggested national standards, British Orthopedic Association Standards for Trauma (BOAST) guidelines for the management of ankle fractures, which also reflect recommendations from National Institute for Health and Care Excellence (NICE) [3]. However, there remains much controversy and variations in practice, especially with regards to the weight-bearing status in both operatively and non-operatively managed ankle fractures [4]. Early weight-bearing in these fractures has been shown to reduce thromboembolic events, improve functional outcomes, early return to work, and or pre-injury status. However, there remains the risk of displacement of fractures resulting in malunion, need for further surgery, and early-onset ankle arthritis [5,6].

Multiple recent studies have utilized the power of trainee research collaborative (TRC) networks to gather data from a large number of patients from hospitals across wide geographical areas in a short period [4,7,8]. These have encouraged high-quality multi-center research to limit bias and increase the external validity of the study results, as well as to nurture a culture of collaboration. Inspired by these successful TRCs, the trainee-led Welsh Orthopedic Research Collaborative (WORC) was created in late 2019 to conduct national audits across Wales and to increase the accessibility of research for orthopedic trainees. The current study is the first of many all Wales national audits run using this network.

The purpose of this study was to audit the practice of orthopedic departments of NHS hospitals across Wales in the management of operatively and non-operatively treated ankle fractures and compliance with published BOAST guidance. Based on the observation by various trainees regarding the management of ankle fractures in different hospitals across Wales, we hypothesized that compliance with BOAST guidance is generally poor, especially regarding early weight-bearing.

## Materials and methods

We performed a multicentre, snapshot, prospective audit of adult (>16 years) ankle fractures between February 2, 2020, and February 17, 2020. Pediatric, pathological, and open fractures were excluded. Collaborators were recruited via the Welsh Orthopedic Research Collaborative (WORC). The audit leads (RA and JC) created a data collection proforma which was then uploaded on Welsh Orthopedic Educational Hub (www.walesortho.co.uk). This is a password-protected website to which all Welsh orthopedic trainees and trainers have access. Regional leads were recruited in nine hospitals across Wales who further recruited participants in their respective hospitals for data collection. After two weeks, data were sent back to the audit leads via the educational hub website. None of the data had any patient identifiers. Research and ethics committee approval was unnecessary as per the Health Research Authority decision tool (www.hra-decisiontools.org.uk/research). All collaborators took permission from local health boards for data collection.

The data collected is shown in Table 1. For questions 1, 5, 6, and 7, the participants were asked to fill all the applicable options. We defined early weight-bearing (EWB) as unrestricted weight-bearing on the affected leg within three weeks of injury or surgery and delayed weight-bearing (DWB) as unrestricted weight-bearing after three weeks of injury or surgery. EWB applied even if the patient was allowed unrestricted weight-bearing in an orthosis or cast. For the operative cases, weight-bearing instructions were observed from the operative note. No pre-operative or postoperative outcome data were collected.

**Table 1:** Data collected for the audit M: medial; L: lateral; P: posterior; MOI: mechanism of injury; NV: neurovascular; WB: weight-bearing; NWB: non-weight-bearing; PWB: partial weight-bearing; NV: neurovascular; op: operative

1	Documentation
1a	MOI
1b	Skin integrity
1c	NV status
1d	Co-morbidities
1e	Alcohol intake
1f	Smoking status
2	Was a true lateral and mortice view done at presentation?
3	Type of fracture
3a	Uni-malleolar: medial/lateral/posterior
3b	Bi-malleolar : M+L, M+P, L+P
3c	Tri-malleolar
3d	Associated syndesmotic injury?: yes or no
4	Was weight-bearing X-ray done in orthopedic clinic?
5	For non-operative group: treatment given
5a	NWB with duration
5b	Unrestricted WB
5b.1	WB in boot
5b.2	WB in cast
6	For op group: type of surgery
6a	Lateral malleolus fixation
6b	Medial malleolus fixation
6c	Posterior malleolus fixation
6d	Syndesmotic fixation
7	Weight-bearing after surgery as per operative note
7a	Initial NWB or PWB with duration
7b	Weight-bearing as tolerated

We used Microsoft Excel for Mac (Redmond, WA: Microsoft) and IBM SPSS Statistics version 20 (Armonk, NY: IBM Corp.). Nominal and ordinal data were summarized as numbers and percentages. The chi-square test was used for the 2x2 contingency table. Significance was defined as p≤0.05.

## Results

A total of 28 collaborators from nine NHS hospitals across six health boards in Wales collaborated data of 238 ankle fractures on 238 patients. Listwise deletion was used to deal with missing data. Data for 12 patients were incomplete and excluded. Table 2 shows the contribution from each hospital. Table 3 shows if the mechanism of injury, skin integrity, neurovascular status, co-morbidities, alcohol intake, and smoking status were documented on initial presentation.

**Table 2:** Data contributed by each hospital N: number

	Operative group (n {%})	Non-operative group (n {%})
Neville Hall	11 (10)	17 (16)
Ysbyty Gwynedd	4 (3.6)	1 (0.9)
Princess Of Wales	15 (13.6)	16 (15.1)
Royal Gwent	27 (24.5)	10 (9.4)
Glangwili Hospital	11 (10)	17 (16)
Morriston Hospital	18 (16.4)	24 (22.6)
Wrexham Maelor	3 (3.7)	4 (3.8)
Royal Alexandra	8 (7.3)	14 (13.2)
University Hospital Wales	13 (11.8)	3 (2.8)

**Table 3:** Data regarding documentation at initial presentation N: number

Criteria	Documentation, yes (n {%})	Documentation, no (n {%})
Mechanism of injury	94.4	5.6
Skin integrity	71.3	28.7
Neurovascular status	80.1	19.9
Co-morbidities	81	19
Alcohol intake	34.3	65.7
Smoking status	41.7	58.3

All patients had a true lateral view at initial presentation but only 75.9% of patients had an anteroposterior (AP) mortice view; 28.19% (31/110) and 8.49% (9/106) had a posterior malleolus fracture (isolated or in combination with other malleoli fractures) in the operative and non-operative group, respectively. Only 15/31 (48.38 %) and 0/9 (0 %) had a CT scan for further evaluation.

The majority of cases in the non-operative group were uni-malleolar fractures, while the majority in the operative group was bi-malleolar (Table 4). Nearly 80% of the patients in the non-operative group did not have a weight-bearing X-ray to assess the stability of the ankle fracture (Table 5). Approximately 40% of the patients treated non-operatively had DWB (Table 6). Of the 22 patients who had a weight-bearing X-ray to rest stability, 15 (68.18%) progressed to EWB, and seven (31.82%) had DWB. Of the 84 patients who did not have X-ray to assess stability, 49 (58.33%) progressed to EWB and 35 (41.67) had DWB. This difference was not statistically significant (x^2^ (2) = 0.36, p = 0.27). 

**Table 4:** Distribution of ankle fractures based on the classification MM: medial malleolus; LM: lateral malleolus; PM: posterior malleolus; n: number

Fracture type	Operative group (n {%})	Non-operative group (n {%})
Isolated MM	1 (0.9)	10 (9.4)
Isolated Weber A LM	0	20 (18.9)
Isolated Weber B LM	3 (2.7)	51 (48.1)
Isolated Weber C LM	3 (2.7)	5 (4.7)
Isolated PM	0	7 (6.6)
Isolated syndesmotic injury	0	0
MM + LM	72 (65.5)	11 (10.4)
MM + PM	0	1 (0.9)
LM + PM	1 (0.9)	1 (0.9)
Tri-malleolar	30 (27.3)	0
Total	110	106
Associated syndesmotic injury	59 (53.6)	2 (1.9)

**Table 5:** Data showing if a weight-bearing X-ray was done in the non-operative group MM: medial malleolus; LM: lateral malleolus; PM: posterior malleolus; NA: not applicable; n: number

Fracture type	Yes (n {%})	No (n {%})
Isolated MM	2 (20)	8 (80)
Isolated Weber A LM	0 (0)	20 (100)
Isolated Weber B LM	15 (29.4)	36 (70.6)
Isolated Weber C LM	2 (40)	3 (60)
Isolated PM	1 (14.3)	6 (85.7)
Isolated Syndesmotic Injury	NA	NA
MM + LM	2 (18.2)	9 (81.8)
MM + PM	0 (0)	1 (100)
LM + PM	0 (0)	1 (100)
Overall	22 (20.8)	84 (79.2)

**Table 6:** Data showing weight-bearing status among the different fracture patterns in the non-operative group MM: medial malleolus; LM: lateral malleolus; PM: posterior malleolus; NA: not applicable; n: number

Fracture type	Early weight-bearing (n {%})	Delayed weight-bearing (n {%})
Isolated MM	4 (40)	6 (60)
Isolated Weber A LM	14 (70)	6 (30)
Isolated Weber B LM	31 (60.8)	20 (39.2)
Isolated Weber C LM	4 (80)	1 (20)
Isolated PM	2 (28.6)	5 (71.4)
Isolated syndesmotic injury	NA	NA
MM + LM	7 (63.6)	4 (36.4)
MM + PM	1 (100)	0 (0)
LM + PM	0 (0)	1 (100)
Overall	63 (59.43)	43 (40.57)

Table 7 shows the type of fixation in the operative group and weight-bearing status as per the operative note. The reason for post-operative delayed weight-bearing was mentioned in 16.16% (16/99) patients only. These were concerns regarding fixation (n = 9), osteoporosis (n = 5), soft tissue concerns (n = 4), and raised BMI (n = 3). Four patients had more than one reason mentioned. Table 8 shows the distribution of weight-bearing status (combined operative and non-operative) among the contributing hospitals.

**Table 7:** Type of fixation and subsequent weight-bearing status MM: medial malleolus fixation; LM: lateral malleolus fixation; PM: posterior malleolus fixation; Syn: syndesmotic fixation; EWB: early weight-bearing; DWB: delayed weight-bearing; n: number

	Total (n {%})	EWB (n {%})	DWB (n {%})
LM + MM	33 (30)	6 (18.2)	27 (81.8)
LM + MM + Syn	20 (18.2)	3 (15)	17 (85)
LM + PM	6 (5.4)	0(0)	6 (100)
LM + Syn	16 (14.6)	1 (6.3)	15 (93.8)
LM Only	12 (10.9)	1 (8.3)	11 (91.7)
MM + MP	1 (0.9)	0 (0)	1 (100)
MM + Syn	3 (2.7)	0 (0)	3 (100)
MM Only	3 (2.7)	0 (0)	3 (100)
PM + Syn	1 (0.9)	0 (0)	1 (100)
Only Syn	6 (5.5)	0 (0)	6 (100)
Tri-malleolar fixation	9 (8.2)	0 (0)	9 (100)
Total	110 (100)	11 (10)	99 (90)

**Table 8:** Distribution of weight-bearing in the contributing hospitals N: number

	Early weight-bearing (n {%})	Delayed weight-bearing (n {%})
Neville Hall	35.71	64.29
Ysbyty Gwynedd	80.00	20.00
Princess Of Wales	25.81	74.19
Royal Gwent	32.43	67.57
Glangwili Hospital	42.86	57.14
Morriston Hospital	42.86	57.14
Wrexham Maelor	28.57	71.43
Royal Alexandra	22.73	77.27
University Hospital Wales	25.00	75.00

## Discussion

Being a trainee-led collaborative, this study collected data from all of the six university health boards in Wales which have a university/district general hospital [9]. Clear and accurate documentation is a part of the good medical practice guidelines issued by the general medical council [10]. Appropriate documentation is an important part of good medical practice and also contributes to a reduction in the number of medicolegal claims [11,12]. Points one and two of the BOAST guidelines clearly state that mechanism of injury, clinical findings, and comorbidities should be clearly documented. In our study, we found that the mechanism of injury was documented in nearly all patients but co-morbidities and neurovascular status were documented in only four out of five patients. There was poor documentation of alcohol and smoking status, both of which can influence the management of these fractures [13,14].

With improvement in understanding of the role of the posterior malleolus in the biomechanics of ankle fractures, further imaging in form of a CT scan is recommended for complex fracture patterns, especially the ones involving posterior malleolus (PM) [15,16]. Our study had a total of 40 posterior malleolar fractures with the majority involving one or more malleoli. However, less than 50% of these were imaged with a CT scan. Interestingly, none of the patients in the non-operative group with a PM fracture received a CT scan. A CT scan is imperative to determine the size and morphology of the PM fragment which will determine its management. Biomechanical studies have shown that the posterior one-fourth of the ankle joint remains largely unloaded [17]. Only one out of the nine patients who had PM fracture in the nonoperative group had a weight-bearing X-ray. A weight-bearing X-ray (WBXR) can potentially show if the fracture is stable and therefore suitable to treat non-operatively with EWB. 

As per BOAST guidelines, fractures considered stable should be allowed to bear weight as tolerated. In the non-operative group, nearly 40% of patients had DWB. Of the 93 uni-malleolar fractures in the non-operative group, only 55 (59%) were treated with early weight-bearing. If ankle stability is uncertain, then weight-bearing radiographs will help to determine this [18]. This is recommended by BOAST as well, however, only 22% of the patients in the non-operative group had a WBXR. If patients had a WBXR, there was an increased chance of being treated with EWB, however, this was not statistically significant. 

BOAST guidelines suggest that most patients should be allowed to bear weight as tolerated in splint or cast after surgery unless there are contraindications or concerns regarding fixation. EWB allows for quicker discharge from the hospital, early return to work, reduces calf atrophy and decreases chances of deep venous thrombosis [5,19]. However, only 10% of the operatively treated ankle fractures had EWB. EWB status was poor across all types of fixation as shown in Table 7. The reason for DWB was mentioned in only 16% of the patients. 

One of the reasons for the low rate of EWB may be the ambiguity regarding the guidelines. The BOAST guidelines state that most operatively treated fractures should be allowed EWB but do not specify if patients with syndesmotic fixation or posterior malleolus fixation can have EWB. This is evident in our study as 14.28% of patients (7 of 49) who did not have an either PM or syndesmotic fixation were allowed EWB but only 6.56% (4 of 61) of those who had PM or syndesmotic fixation were allowed EWB. This was, however, not statistically significant (Fisher's exact test, p = 0.15). 

Most of the ankle fixation surgeries (except complex patterns) are performed by trainees or general trauma surgeons. Anecdotally, we feel that there is a fear of causing failure of fixation or wound complications by allowing EWB. Multiple studies do recommend EWB even in presence of syndesmotic fixation or PM fracture [5,6,15,18,19]. However, most of these studies are single-center case series. Two large multicenter randomized controlled trials are currently in process which may provide the definitive answer to weight-bearing status after fixation of ankle fractures [20,21].

The results of this study are poor as compared to a recent trainee collaborative study on ankle fractures [4]. In our study, 60% and 10% of patients were allowed EWB in the non-operative and operative group, respectively, as compared to 81% and 21% in the other study. This will have significant financial implications as well because DWB can contribute to an increase in hospital stay and loss of wages [22]. DWB also has a negative effect on patient satisfaction and psychology. Patients describe having to "endure" the period of non-weight bearing and avoid simple but important daily tasks like bathing or showering until full weight bearing is permitted. 

This study demonstrates the ability of the Welsh Orthopedic Research Collaborative to complete multicentre audits quickly, which will allow future projects within the deanery to be completed with a significantly larger patient sample than would be possible in single-center studies. Working as a collaborative will also give us opportunities to cooperate with other similar regional units to produce high-quality data from trainee-led studies.

Regarding this study, following dissemination of results across Wales, we plan to work with the regional leads to promote education regarding ankle fracture management and BOAST guidelines within their departments. We also aim to make a simple infographic sheet that will aid in the management of ankle fractures. We aim to complete a re-audit within the next academic year to see whether compliance has improved.

We acknowledge the limitations of this study. We have reported the decisions made by the treating surgeons but the justification behind the decisions has not been recorded. We have not collected any outcome measures or any complications in both groups as the aim of this study was to check compliance against BOAST guidelines. We have not collected data of any cross-over from non-operative to operative treatment. 

## Conclusions

This study demonstrates overall poor compliance with BOAST guidelines across Wales. We need to be better at the documentation and also need to consider performing a CT scan when the posterior malleolus is fractured. A weight-bearing X-ray should be performed more often to ascertain the stability of an ankle fracture and those that are deemed stable should be treated with early weight-bearing. The guidelines need to be clearer regarding weight-bearing after fixation, especially when the posterior malleolus and/or syndesmosis are stabilized.
